# An analysis of the association between workplace intergenerational mentoring characteristics and employees’ receptivity to change: the mediating role of contextualized human capital perception

**DOI:** 10.3389/fpsyg.2026.1899021

**Published:** 2026-07-27

**Authors:** Chunli Sun, Yange Qi

**Affiliations:** 1 School of Economics and Trade Management, Zhengzhou Institute of Technology, Zhengzhou, China; 2 School of Arts and Education, Zhengzhou Institute of Technology, Zhengzhou, China

**Keywords:** contextualized human capital perception, intergenerational mentoring, multigenerational employees, organizational change, receptivity to change, self-perceived employability

## Abstract

**Inroduction:**

With the normalization of organizational change and the “multi-generational coexistence” of the labor market, understanding and mitigating employees’ resistance to change has become increasingly important. Based on a contextualized perceived human capital perspective, this study aimed to explore the associations among intergenerational mentoring characteristics, employees’ subjective appraisal of their career-related human capital, and organizational change receptivity.

**Methods:**

Utilizing a cross-sectional design with self-report surveys, data were collected from 812 active employees across various industries. The study evaluated intergenerational mentoring interactions, contextualized human capital perception (operationalized through self-perceived employability indicators that capture perceived career competitiveness, skill transferability, and marketability), and organizational change receptivity.

**Results:**

The empirical results demonstrate that: (1) Intergenerational mentoring interactions (including career support, psychosocial support, and role modeling) are significantly and positively correlated with employees’ receptivity to change; (2) contextualized human capital perception presents a significant partial statistical mediation association in the relationship between intergenerational mentoring and change receptivity (indirect effect *B* = 0.171, 95% CI [0.135, 0.206], accounting for 25.2% of the total statistical effect); (3) no significant between-group differences in change receptivity were found among employees of different birth generations, rank levels, or industries. However, these non-significant demographic findings should be interpreted cautiously given unequal group sizes, especially the relatively small senior-generation group.

**Discussion:**

These findings suggest that cross-generational support networks are positively associated with employees’ evaluations of their own employability, which is correlatively linked to a more favorable attitude toward change. Because the study relies on cross-sectional self-report data, the observed mediation should be strictly interpreted as a statistical association rather than a causal mechanism. Future longitudinal and multi-source studies are necessary for further validation.

## 1 Introduction

In the current business environment, intensified external market competition and rapid technological iteration have made organizational change no longer merely an occasional response to crisis but increasingly a routine condition for organizational survival and development (Burnes et al., 2018; Hanelt et al., 2021). Whether introducing digital systems or optimizing organizational structures, these changes profoundly affect employees’ work patterns and psychological contracts. At the same time, the labor market presents a significant feature of “multi-generational coexistence” (Alaql et al., 2023), and employees of different age groups differ in career stages, experience, and tolerance for uncertainty. This difference often traps management practices into fixed generational stereotypes such as “older employees are naturally more resistant to change” (Wang and Duan, 2025). Facing the anxiety and resistance caused by normalized change, existing research mostly explores the formation mechanism of change attitudes from top-down perspectives such as leadership, organizational communication, or institutional design, but often ignores the most intensive micro-informal interactions in the workplace. Therefore, internal support networks in the workplace, especially intergenerational mentoring, provide a practically significant entry point for alleviating resistance to change. Intergenerational mentoring not only includes traditional mentoring where senior employees pass on experience to young employees, but also covers reverse support provided by young employees on digital tools and new working methods (Browne, 2021; Marcinkus Murphy, 2012; Venugopal et al., 2025). Whether this cross-age work coaching, emotional comfort, and cognitive communication can promote smooth acceptance of organizational change by employees of different generations holds important empirical testing value.

To explore the psychological deduction path between intergenerational mentoring characteristics and receptivity to change, this study introduces and deepens the human capital perspective at the micro level (Becker, 1964). Traditional human capital is often viewed as an objective educational background or skill stock. However, in organizational psychology, employees’ responses to uncertainty depend not only on the objective resources they possess, but also on their subjective appraisal of whether these resources are useful, transferable, and sufficient for coping with career risks. Therefore, this study distinguishes objective human capital from contextualized human capital perception. The latter refers to employees’ perceived value, transferability, and marketability of their career-related knowledge, skills, experience, and learning capacity in the context of organizational change. Intergenerational guidance and communication in the workplace can be regarded as a key resource supply channel, which may be associated with a lower sense of change threat and a higher level of receptivity to change through employees’ more positive perception of their own career competitiveness and employability-related human capital.

In summary, this study aims to examine the relationship between workplace intergenerational mentoring interactions, employees’ human capital perception, and organizational change receptivity, and focuses on testing the mediating role of human capital perception therein. Furthermore, given the stereotype in management practice that “older employees are more resistant to change” or “younger employees are more likely to accept change,” this study will also exploratively test whether there are significant differences in receptivity to change among employees of different birth generations, rank levels, and industries. This study integrates intergenerational support and individual resource perception into the same research framework, which not only expands relevant theories of the micro-mechanisms of organizational change and multi-generational employee management, but also provides empirical evidence for enterprises to achieve flexible change management by building cross-generational cross-mentoring networks when implementing technological transformation and institutional restructuring. Given the cross-sectional design, this study focuses on theoretically grounded statistical associations rather than establishing temporal or causal ordering among the variables.

## 2 Theoretical basis and research hypotheses

### 2.1 Conceptual definitions and literature review

#### 2.1.1 Workplace intergenerational mentoring and its characteristics

Mentoring generally refers to a supportive work relationship established between an experienced person and a relatively inexperienced person in the workplace. According to research by Noe (1988) and Scandura and Ragins (1993), mentoring support typically encompasses three dimensions: career support (e.g., skill transfer, resource referral), psychosocial support (e.g., emotional understanding, acceptance), and role modeling (e.g., demonstration of work attitudes and problem-solving approaches).

With the change in the intergenerational structure of the workplace, intergenerational mentoring is no longer limited to the one-way “old leading new.” In this study, intergenerational mentoring is defined as: an instructional interactive process formed around skill transfer, psychological support, and behavioral demonstration between employees of different birth generations in the workplace. Whether it is senior employees passing on business experience to young employees, or young employees sharing digital tools and new working methods with older colleagues (Bertram et al., 2023; Foliot and Chaliès, 2025), both are included in the measurement framework of this paper. Thus, intergenerational mentoring reflects the two-way flow of knowledge, experience, and emotional resources in informal workplace interactions (Igoa-Iraola and Díez, 2024; Kordova et al., 2022; Madhavanprabhakaran et al., 2022; Tang and Martins, 2021).

#### 2.1.2 Receptivity to organizational change

Attitudinal receptivity to change refers to employees’ tendency to accept, support, and cooperate when facing new systems, new structures, or new business processes implemented by the enterprise (Khaw et al., 2023). Based on the three-component attitude model of Oreg (2006) and Herscovitch and Meyer’s (2002) research on commitment to change, this receptivity is not unidimensional, but a comprehensive manifestation of cognition, emotion, and behavioral intention. Cognitive receptivity is manifested as employees’ recognition of the necessity and long-term value of the change; emotional receptivity is manifested as the emotional responses formed by employees to uncertainty, potential losses, and adaptation pressure during the change process, including emotional states such as anxiety, worry, optimism, or hope, the core of which lies in whether employees can face the change process relatively steadily on an emotional level; behavioral receptivity reflects employees’ willingness to follow new processes, learn new skills, and actively participate in the implementation of the change. Accordingly, this study defines organizational change receptivity as: a comprehensive attitude in which employees, based on an understanding of the necessity of organizational change, remain emotionally relatively stable and show willingness to cooperate and participate in behavior.

#### 2.1.3 Conceptual connotation and psychological representation of human capital perception

In macroeconomics and management, Human Capital generally refers to the sum of objective resources such as knowledge and skills possessed by individuals (Becker, 1964). However, objective human capital and employees’ subjective perception of that capital should be analytically distinguished. Employees with similar education, tenure, or skill portfolios may differ substantially in whether they believe these resources are useful, transferable, and sufficient for coping with organizational turbulence. Therefore, in the micro-field of organizational behavior, how employees respond to uncertainty depends not only on the objective resources they possess, but also on their subjective appraisal of the career value and usability of these resources. Accordingly, this study shifts the focus from “objective stock” to “subjective perception,” namely “human capital perception.”

In operational definition, this study understands human capital perception as employees’ subjective appraisal of the usefulness, transferability, and career value of their knowledge, skills, experience, and learning capacity in the context of organizational change. This construct is conceptually distinct from several adjacent concepts. First, it differs from objective human capital, which refers to externally observable resources such as education, qualifications, certificates, and accumulated work experience. Second, it differs from general adaptability confidence, which emphasizes confidence in adjusting to new situations. Third, it differs from change-related self-confidence or change self-efficacy, which focuses more narrowly on confidence in handling a specific change event. By contrast, contextualized human capital perception concerns whether employees believe their broader career-related resources remain valuable and transferable both inside and outside the organization.

To measure this construct, this study adopted the “self-perceived employability” proposed by Rothwell and Arnold (2007). This does not mean that perceived employability is identical to human capital itself. Rather, perceived employability is used as an operational indicator of employees’ subjective evaluation of whether their human capital can be converted into internal career security and external labor-market competitiveness. This operational logic is grounded in classic organizational psychology literature. According to the widely recognized psychosocial construct of employability proposed by Fugate et al. (2004), human capital constitutes the inseparable and foundational dimension of an individual’s employability. Furthermore, Forrier et al. (2015) point out that objective human capital only translates into psychological security when it is cognitively activated and appraised by the individual as functional “movement capital” or “marketability.” The reason why these two can be theoretically self-consistent is that in the contemporary volatile employment relationship, employees’ perception of their own employability (Mainga et al., 2025) is precisely the most direct reflection of whether their human capital can effectively cope with market fluctuations and organizational change (Ho et al., 2023; Jackson et al., 2024; Lo Presti et al., 2022).

Therefore, in the organizational change context of this study, perceived employability does not replace the concept of human capital, but serves as a contextually appropriate operational proxy for human capital perception when facing workplace threats. It essentially represents the individual’s subjective and dynamic psychological mapping of their human capital’s utility (Van der Heijden and Van der Heijden, 2006). When employees feel strong career competitiveness and external market value in cross-generational interactions, it marks that they have completed a positive confirmation of their human capital reserves. Nevertheless, because the empirical measurement relies on self-perceived employability indicators, the findings of this study should be interpreted as evidence concerning perceived career competitiveness and employability-related human capital perception, rather than as evidence concerning objective human capital itself.

### 2.2 Research hypotheses

Based on the above conceptual definitions, this study further deduces the relationships between variables combining social exchange theory and conservation of resources theory. These three perspectives play complementary rather than parallel roles in the present framework. Human capital theory provides the primary lens for understanding why employees’ perceived career resources matter in uncertain organizational environments. Social exchange theory explains why intergenerational mentoring, as an informal support relationship, may be associated with more cooperative and positive workplace attitudes. Conservation of resources theory further clarifies why employees who perceive stronger career-related resources may appraise organizational change as less threatening. Thus, human capital theory defines the focal psychological resource, social exchange theory explains the relational source of that resource, and conservation of resources theory explains why perceived resource sufficiency is associated with receptivity to change. Given that this study uses a cross-sectional questionnaire design, the following hypotheses all point to statistical association mechanisms between variables, rather than strict causal relationships.

#### 2.2.1 The relationship between intergenerational mentoring characteristics and receptivity to change

Faced with structural or systemic changes implemented by organizations, employees are often prone to exhibiting uncertainty and ability anxiety. Social exchange theory (Blau, 1964; Cropanzano and Mitchell, 2005) points out that when employees feel support from colleagues or other members within the organization, they are more likely to show positive cooperation willingness. In a multi-generational interacting workplace, mentoring support functions can provide employees with composite resources: career and skill support provides a cognitive basis for employees to understand new systems, which is often accompanied by lower cognitive resistance; psychosocial support shows a negative association with anxiety during the change process through emotional resonance; role modeling provides employees with referable adaptation strategies. Therefore, higher-quality intergenerational mentoring interactions often show a co-occurrence relationship with higher levels of organizational change receptivity. Accordingly, the hypothesis is proposed:

*H1*: Intergenerational mentoring characteristics are significantly and positively correlated with employees’ organizational change receptivity.

#### 2.2.2 The relationship between intergenerational mentoring characteristics and human capital perception

From the perspective of workplace learning, cross-generational interaction is an informal channel for experience transfer and tacit knowledge sharing. The organizational rules and complex problem-solving experience passed on by senior employees, and the digital tools and new technology applications shared by young employees, form a two-way resource exchange. This exchange expands employees’ cognitive boundaries. When employees continuously acquire knowledge and psychological support in interactions, it is often accompanied by a higher-level positive assessment of their own adaptability and career competitiveness (Malik and Nawaz, 2022). Therefore, cross-generational interactive support networks are closely related to employees’ positive self-resource cognition. Accordingly, the hypothesis is proposed:

*H2*: Intergenerational mentoring characteristics are significantly and positively correlated with employees’ human capital perception.

#### 2.2.3 The relationship between human capital perception and receptivity to change

Conservation of resources theory (Hobfoll, 1989) argues that individuals have a tendency to acquire, maintain, and protect their own resources. When external environmental changes are accompanied by potential risks of resource loss, individuals are prone to show defense or resistance; conversely, individuals with abundant resource reserves often show a more active intervention posture when facing environmental changes. In organizational change, human capital perception is the core psychological resource. Existing research shows that employee ability perception and organizational context factors affect their work stress experience, and when employees believe their abilities are sufficient to cope with job demands, their stress responses are often lower (Kim and Jung, 2022). Therefore, when employees perceive themselves as having strong learning ability, position adaptability, and external employability, they tend to have lower threat appraisals when facing organizational change, and are more likely to view system adjustments or process restructurings as opportunities to accumulate career capital, thereby showing higher cognitive recognition and behavioral cooperation willingness. Accordingly, the hypothesis is proposed:

*H3*: Employees’ human capital perception is significantly and positively correlated with organizational change receptivity.

#### 2.2.4 The statistical mediation association of human capital perception

Synthesizing the logic of H1–H3, this study speculates that there is an indirect statistical path connecting intergenerational mentoring characteristics and receptivity to change via human capital perception. Specifically, cross-generational interactions are not only directly manifested as skill and emotional support in change contexts, but also associated with employees’ abundant perception of resources such as their own career competitiveness; and this resource state based on ability confidence further tallies with a more positive attitude toward change receptivity. In other words, employees’ positive acceptance of change, in a statistical sense, not only reflects the existence of external support networks but also reflects the abundant state of individual internal ability resources. Human capital perception acts as a key bridge in this. Accordingly, the hypothesis is proposed:

*H4*: Human capital perception presents a significant statistical mediation association between intergenerational mentoring characteristics and organizational change receptivity.

#### 2.2.5 Exploratory question of birth generation differences

In multi-generational workplace research, traditional management experience often carries generational stereotypes such as “older employees are more resistant to change” or “younger employees are more likely to accept change” (Wang and Duan, 2025). However, with the generalization of employment uncertainty and performance pressure, employees of different age groups may show similar resource protection tendencies when facing changes involving job security. Therefore, this paper does not presuppose that specific generational groups are tied to higher resistance to change, but objectively tests birth generation differences as an exploratory question.

RQ1: Are there significant between-group differences in the overall scores of organizational change receptivity among employees of different birth generations?

**Figure 1:**
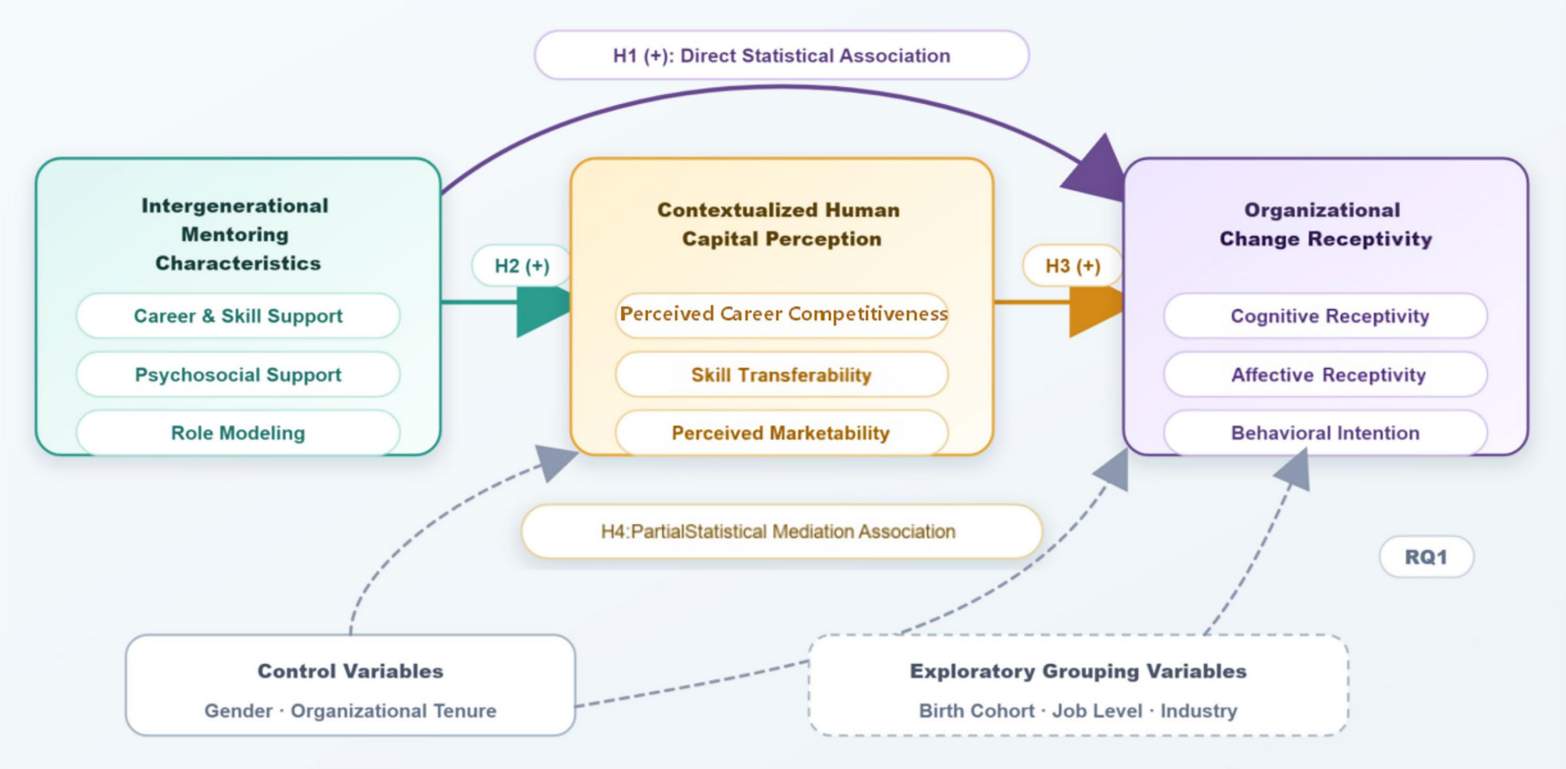
Theoretical model of workplace intergenerational mentoring, contextualized human capital perception, and organizational change receptivity. H1–H4 represent the hypotheses proposed in this study; RQ1 represents the exploratory research question; Solid arrows indicate hypothesized statistical associations among the core variables. Dashed lines indicate the paths of control variables or exploratory grouping comparisons. Given the cross-sectional research design, the arrows represent theoretically derived statistical associations rather than confirmed causal effects.

Based on the above theoretical derivations, this study constructs a theoretical model among workplace intergenerational mentoring characteristics, human capital perception, and organizational change receptivity, as shown in Figure 1. The model takes intergenerational mentoring characteristics as the core independent variable, organizational change receptivity as the outcome variable, and introduces contextualized human capital perception as the mediating variable to explain how cross-generational mentoring interactions form a statistical association with a higher level of change receptivity through employees’ positive perceptions of their own career competitiveness, employability, and job adaptability. At the same time, the model includes gender and tenure as control variables, and uses birth generation, rank level, and industry as exploratory grouping variables to test potential differences in receptivity to change among different employee groups.

## 3 Research methodology and design

### 3.1 Research sample and data collection process

Between January and March 2026, this study relied on an online questionnaire platform to target the multi-industry active practitioner group via social networks, adopting a one-time cross-sectional design for data collection, aiming to capture employees’ self-reported attitudes and subjective perceptions regarding a current, recent, or salient past organizational change. Because a complete sampling frame of multi-industry active employees was not available, this study used a non-probability convenience sampling strategy combined with snowball recruitment. The questionnaire link was distributed through professional social networks, alumni and workplace communication groups, and personal professional contacts. Participants were encouraged to forward the questionnaire only to individuals who were aged 18 or above and currently employed.

To comply with basic research ethics, an informed consent statement was presented on the first page of the questionnaire. Participation was voluntary and anonymous, and respondents were informed that the data would be used only for academic research. Participation was restricted to respondents who confirmed that they were at least 18 years old and currently employed. Respondents were also asked whether their organization had experienced or was currently undergoing significant management or system changes in the past year. Those who answered “No” were instructed to answer the change-related items based on the most salient organizational change experience in their previous career, and this information was recorded as the variable ChangeExp.

To mitigate potential common method bias at the procedural level, several design measures were adopted. Respondents were informed that there were no right or wrong answers and were encouraged to answer honestly. Items measuring different constructs were arranged in separate questionnaire sections, neutral wording was used where possible, and reverse-scored items were included in the change receptivity scale to reduce automatic response patterns.

A total of 860 initial questionnaires were collected in this online survey. Before formal analysis, this study performed a data cleaning procedure, as described in Section 4.1, and finally retained 812 valid responses for statistical analysis. Overall, the final sample included respondents from multiple major industries relevant to digital transformation and process restructuring, such as manufacturing and engineering, Internet and communication, finance, healthcare, retail and services, and other sectors. It also covered all four predefined birth cohorts, including pre-1979, post-80s, post-90s, and post-00s employees. This cross-industry and multi-generational sample structure provided a heterogeneous empirical basis for subsequent theoretical model testing and preliminary exploratory group comparisons.

However, because participation was voluntary and online recruitment was used, potential self-selection bias cannot be fully ruled out. Moreover, given the non-probability sampling strategy and unequal subgroup sizes, the generalizability of the findings should be understood as applying primarily to comparable active employees in multi-industry Chinese workplace contexts.

### 3.2 Measurement instruments and variable constructs

This study mainly refers to mature scales in the fields of organizational behavior and psychology, and makes semantic adjustments combined with the Chinese workplace and organizational change context. The questionnaire items were processed through a Chinese-English back-translation procedure to ensure semantic equivalence, and uniformly scored using a 5-point Likert scale (1 means “strongly disagree,” 5 means “strongly agree”). The complete items are detailed in Supplementary material 1: Survey Questionnaire. The specific measurement details are as follows:

#### 3.2.1 Independent variable: intergenerational mentoring characteristics

This study refers to the classic division of mentoring functions by Noe (1988) and Scandura and Ragins (1993), and makes wording adjustments combined with cross-generational interaction and reverse mentoring contexts, containing 15 items in total. The scale covers three sub-dimensions: the first is career and skill support, measuring the mutual sharing of system experience and professional skills among colleagues (e.g., “He/She often shares with me the professional skills to complete a specific task or use a new system”); the second is psychosocial support, measuring the emotional understanding and acceptance tendency brought by cross-generational colleagues (e.g., “Despite differences in age/generation, he/she can still understand my emotions and pressure in the workplace”); the third is role modeling, assessing the respondent’s recognition and reference degree of the professional attitudes and problem-solving methods of cross-generational colleagues (e.g., “His/Her behavior provides a good reference point for my future workplace performance”).

#### 3.2.2 Dependent variable: receptivity to organizational change

This variable mainly refers to Oreg’s (2006) three-component attitude framework and Herscovitch and Meyer’s (2002) commitment to change research, forming 15 items combined with the organizational change context, which also contains three sub-dimensions: cognitive receptivity focuses on employees’ recognition of the necessity and long-term value of change (e.g., “I think carrying out this change is necessary and beneficial for the future development of the enterprise”); emotional receptivity focuses on a steady mindset and the reduction or absence of anxiety when facing change (e.g., “Facing this organizational change, I generally feel optimistic and hopeful”); behavioral intention evaluates employees’ willingness to follow new processes and learn new skills (e.g., “I am willing to invest my personal time to learn the new skills required for this change”). To ensure measurement rigor and detect response bias, this scale entails three items that require reverse scoring during data processing.

#### 3.2.3 Mediating variable: contextualized human capital perception

This variable refers to the theoretical connotation of self-perceived employability by Rothwell and Arnold (2007) and has been contextually adapted in combination with the intergenerational interaction context, containing 5 items in total. The scale aims to reflect employees’ subjective evaluation of their own career competitiveness, skill transferability, and perceived market value against the background of change (representative items such as: “The new skills I have mastered increase my value in the external talent market”; “Cross-generational communication broadens cognitive boundaries and makes thinking more agile”).

It should be heavily emphasized that this indicator does not directly measure objective human capital, such as the exact mathematical number of skills, formal credentials, certificates, or years of experience. Instead, it assesses employees’ perceived career-resource value, namely whether they believe their accumulated knowledge, skills, and learning capacity can be transformed into internal adaptability and external employability. Because several items are contextualized to organizational change and intergenerational communication, this measure is operationalized through self-perceived employability indicators. Therefore, throughout the empirical analysis, the interpretation of results is uniformly founded on “contextualized human capital perception” rather than objective human capital itself.

### 3.3 Data processing and control variable settings

Before formally conducting statistical validation, this study made the following processing plans for each variable and prior data information:

First, in defining organizational change experience, the contextual assessment benchmark of the dependent variable is based on the organizational change the respondent is “currently experiencing or has recently experienced within the past year.” For samples lacking recent change experience, the study required them to answer based on the most memorable change in their past career to ensure all samples have an evaluation target, which was strictly controlled as a contextual tracking variable (ChangeExp) during data entry.

Second, in setting target objects for between-group difference testing, to guarantee relatively balanced group sample sizes and facilitate macro comparison, this study uniformly recoded “first-line management, middle management, and top management” in the original data as “management,” and tested for between-group differences with “grassroots employees” who do not lead teams.

Finally, in excluding confounding effects, to prevent characteristic differences in basic demographic dimensions from interfering with the core model, this study uniformly included respondents’ gender and current tenure as control variables in the subsequent hierarchical regression and structural mediation analysis frameworks.

## 4 Data analysis and results display

### 4.1 Data preprocessing and sample basic characteristic analysis

#### 4.1.1 Data cleaning

A total of 860 initial questionnaires were collected in this study. A rigorous data cleaning procedure was executed before formal analysis: first, based on the screening question, only respondents who checked “aged 18 and above and currently employed” were retained, removing 15 non-compliant samples; second, 20 low-quality questionnaires with “straight-line responses” and 13 samples with missing key information were identified and removed. Finally, 812 valid samples were retained, yielding a valid response retention rate of 94.42%. To ensure the accuracy of composite scoring, all reverse-worded items (i.e., Change_CR5_R, Change_ER4_R, and Change_ER5_R) were properly recoded using a reverse-scoring conversion (i.e., 6 minus the original score) prior to subsequent data aggregation. Naming and assignment rules for relevant variables are detailed in Supplementary material 2: Data Codebook.

#### 4.1.2 Distribution of sample demographic characteristics

The demographic characteristics of the valid sample are shown in Table 1.

**Table 1:** Basic demographic characteristics of the survey sample (*N* = 812).

Demographic variable	Category classification	Frequency (*N*)	Percentage (%)
Gender	Male	390	48.0
	Female	422	52.0
Current rank	Grassroots Employee (No subordinates)	446	54.9
	Management (First-line / Middle / Top)	366	45.1
Birth generation	1. Senior Generation (≤1979)	36	4.4
	2. Post-80s (1980–1989)	271	33.4
	3. Post-90s (1990–1999)	379	46.7
	4. Post-00s New Gen (≥2000)	126	15.5
Industry	1. Manufacturing and Engineering	247	30.4
	2. Internet and Communication Tech	209	25.7
	3. Retail Services and Consumer Goods	127	15.6
	4. Finance and Investment Capital	116	14.3
	5. Medical and Pharmaceutical Health	60	7.4
	6. Other Industries	53	6.5

Percentages are calculated based on valid sample *N* = 812, and some percentages may have a 0.1% total error due to rounding.

The data in Table 1 shows that the sample of this study well covers multi-generations, multiple industries, and different rank levels. From the perspective of birth generation, the post-80s and post-90s account for 80.1% in total, forming the backbone of the current workplace and the sample of this study; at the same time, the inclusion of the post-00s new generation (15.5%) provides a rich data basis for exploring the multi-generational workplace context. From the perspective of industry distribution, manufacturing and engineering (30.4%) and Internet and communication technology (25.7%) together account for more than half, indicating that the sample mainly comes from industrial fields where digital transformation and process restructuring pressures are currently most significant. In terms of rank, the proportion of grassroots employees and management is relatively balanced (54.9% vs. 45.1%), providing a sufficient sample demographic basis for subsequent heterogeneity tests.

**Figure 2:**
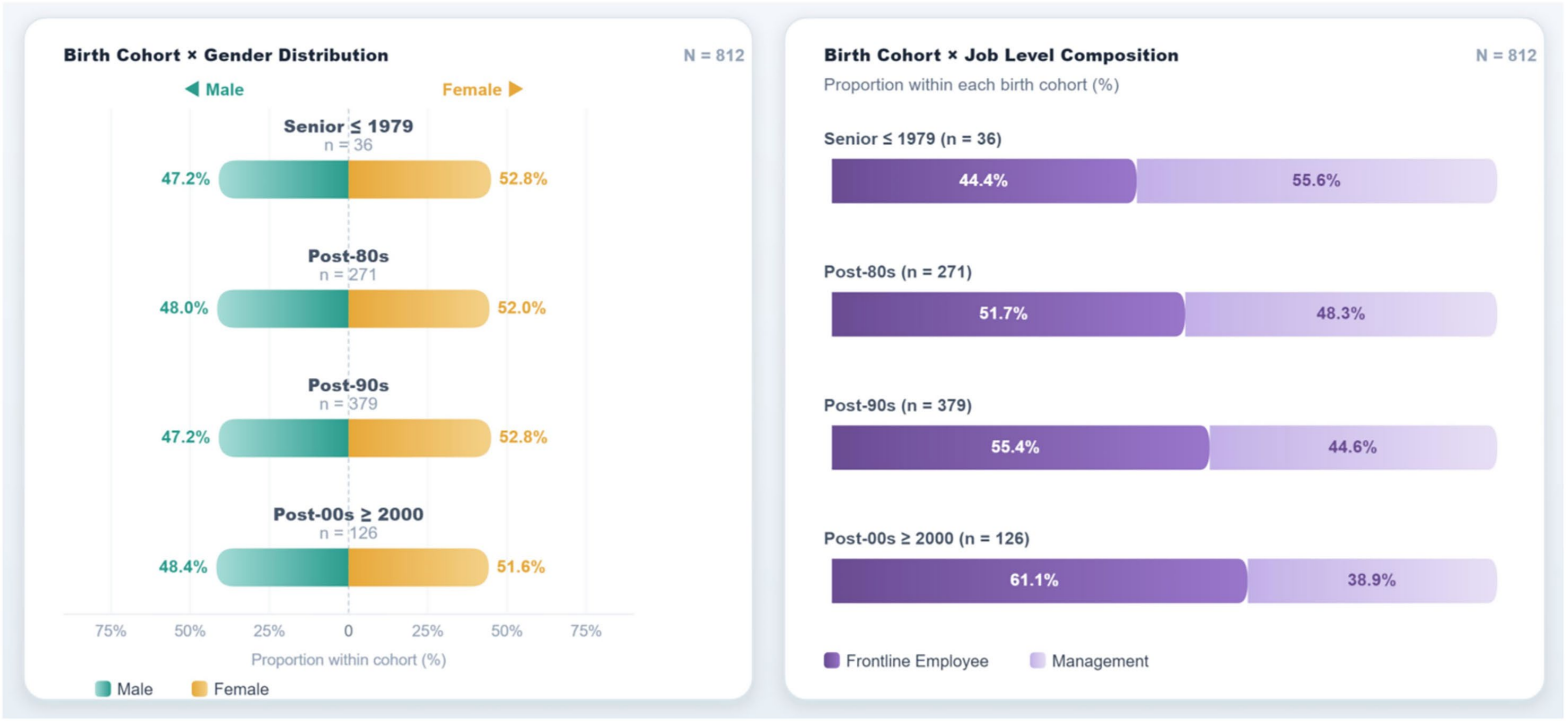
Cross-comparison of gender composition and rank distribution by birth generation (*N* = 812). The left figure shows the gender ratio within each birth generation, and the right figure shows the rank composition within each birth generation. Percentages are calculated based on the sample size within each birth generation.

To further examine whether there is an obvious structural skewness within the sample, Figure 2 conducts a cross-description of gender composition and rank distribution from the perspective of birth generation. The results show that the gender ratio within each birth generation is relatively close, with males accounting for about 47–48% and females accounting for about 52%, indicating that the sample has no obvious gender skewness at different generational levels. At the same time, the rank distribution presents clear generational differences: as the birth generation tends to be younger, the proportion of grassroots employees gradually rises, with the highest proportion of grassroots employees in the post-00s new generation. This structural characteristic is basically consistent with the law of position promotion from the perspective of the career life cycle, that is, younger generations are usually still in the early stages of their careers, and the proportion promoted to management is relatively low. Overall, the cross-distribution shown in Figure 2 further illustrates that the sample of this study has good generational coverage, and also provides a sample structural basis for subsequent multi-generational heterogeneity tests.

### 4.2 Common method bias test and normality test

#### 4.2.1 Common method bias (CMV) test

Because all variables were collected from the same respondents through a cross-sectional self-reported questionnaire, common method variance may be a potential concern. Following Podsakoff et al. (2003), this study addressed common method bias through both procedural and statistical approaches. Procedurally, as described in Section 3.1, the questionnaire was anonymous, respondents were assured that there were no right or wrong answers, the measurement sections for different constructs were separated, and reverse-scored items were included in the change receptivity scale.

Statistically, this study adopted Harman’s single-factor test to conduct unrotated exploratory factor analysis on all measurement items, as shown in Table 2.

**Table 2:** Common method bias diagnostic results.

Diagnostic method	Result	Criterion	Evaluation
Harman’s single-factor test	First factor explained 26.229%	<40%	Acceptable
Full collinearity VIF: Intergenerational mentoring	1.891	<3.3	Acceptable
Full collinearity VIF: Human capital perception	1.585	<3.3	Acceptable
Full collinearity VIF: Receptivity to change	2.063	<3.3	Acceptable

The test results in Table 2 show that the variance explanation rate of the extracted first principal factor is 26.229%, which is significantly lower than the conventional judgment standard of 40%. This indicates that the data in this study does not present a phenomenon dominated by a single factor. However, Harman’s single-factor test is only a preliminary diagnostic. Therefore, this study further conducted a full collinearity variance inflation factor (VIF) test following Kock’s (2015) recommendation. Each core latent variable was regressed on the remaining core latent variables, and the resulting full collinearity VIF values ranged from 1.585 to 2.063. Because all values are well below the conservative threshold of 3.3, severe common method bias is unlikely to fully account for the observed relationships. Nevertheless, because no theoretically unrelated marker variable was included in the questionnaire, residual common method variance cannot be completely excluded.

#### 4.2.2 Descriptive statistics and data feature trends

The descriptive statistics and normality distribution test results for each core variable are shown in Table 3.

**Table 3:** Descriptive statistics and normality test of core variables (*N* = 812).

Variable Name	Mean (*M*)	Std. Deviation (*SD*)	Skewness	Kurtosis
Intergenerational mentoring (Mentor)	3.698	0.539	−0.412	0.301
Human capital perception (HC)	3.753	0.672	−0.528	0.445
Receptivity to change (Change)	3.648	0.428	−0.198	0.122

As can be seen from Table 3, the absolute values of skewness and kurtosis of each variable all meet the statistical requirements for approximate normal distribution. In terms of descriptive characteristics, the average scores of intergenerational mentoring characteristics and human capital perception are *M* = 3.698 and *M* = 3.753, respectively, indicating that respondents generally experienced good cross-generational support in the workplace and maintained a relatively positive assessment of their own career competitiveness. By comparison, the mean value of receptivity to change is *M* = 3.648, reflecting that employees in this sample generally display a moderately high level of positive acceptance when facing organizational changes that involve their own interests or process adjustments.

### 4.3 Reliability and validity test of the measurement model

#### 4.3.1 Reliability and item parceling processing

This study first used Cronbach’s *α* coefficient to evaluate the internal consistency of the scales. The results showed that the α coefficient for intergenerational mentoring characteristics was 0.848, for organizational change receptivity was 0.850, and for human capital perception was 0.764, all higher than the recommended threshold of 0.70, indicating that the measurement tools used in this study have acceptable reliability.

Prior to parceling, because item parceling may conceal multidimensionality, problematic items, or cross-loadings, this study purposefully validated the item-level measurement structures. Specifically, item-level confirmatory factor analyses using maximum likelihood estimation showed that the theoretically predefined three-factor item-level model for intergenerational mentoring (15 items) yielded excellent fit (*χ*^2^/*df* = 1.62, RMSEA = 0.028, CFI = 0.985, TLI = 0.982, SRMR = 0.035). Similarly, the three-factor item-level model for organizational change receptivity (15 items) also demonstrated strong fit properties (*χ*^2^/*df* = 1.25, RMSEA = 0.018, CFI = 0.998, TLI = 0.998, SRMR = 0.026). The standardized factor loadings for all retained items uniformly exceeded the customary 0.50 threshold.

Given that intergenerational mentoring characteristics and receptivity to change contain many measurement items and have pre-confirmed theoretical dimensions, this study conducted Item Parceling referring to the suggestions of Bandalos (2002) and Little et al. (2002). This strategy preserved the substantive meaning of each theoretical dimension while reducing model complexity and estimation instability. Specifically, intergenerational mentoring characteristics and receptivity to change were mean-parceled according to their three intrinsic dimensions, respectively, to form observational indicators; human capital perception retained 5 original items as observational indicators, thereby optimizing model complexity on the premise of ensuring the completeness of construct connotations. Nevertheless, the structural results based on parceled indicators should be interpreted together with the awareness that parceling may absorb some item-level residuals.

#### 4.3.2 Convergent validity and structural validity after dimension reduction

After adopting the item parceling strategy, the convergent validity of the measurement model is generally at an acceptable level. As shown in Table 4, the Average Variance Extracted (AVE) for intergenerational mentoring characteristics is 0.619, and the Composite Reliability (CR) is 0.831; the AVE for organizational change receptivity is 0.584, and the CR is 0.837. For contextualized human capital perception, the AVE is 0.485, marginally lower than the common benchmark of 0.50, while its CR is 0.725, exceeding the recommended threshold of 0.70. Following Fornell and Larcker’s (1981) logic, a marginally lower AVE can still be considered acceptable when composite reliability is adequate and the construct has clear theoretical relevance. Nevertheless, this result suggests that the convergent validity for this contextually adapted mediator should be interpreted cautiously.

**Table 4:** Reliability and validity test results of main variables (*N* = 812).

Variable name	Obs./Parceled indicators	Cronbach’s *α*	Composite reliability (CR)	Average variance extracted (AVE)
Intergenerational mentoring	3 (Parceled indicators)	0.848	0.831	0.619
Human capital perception	5 (Obs. indicators)	0.764	0.725	0.485
Receptivity to change	3 (Parceled indicators)	0.850	0.837	0.584

Intergenerational mentoring characteristics and organizational change receptivity are parceled according to theoretical sub-dimensions; human capital perception retains 5 original items as observational indicators. CR stands for Composite Reliability, AVE stands for Average Variance Extracted.

Subsequently, this study used AMOS software to conduct Confirmatory Factor Analysis (CFA) on the parceled model to examine the structural robustness of the scales (see Table 5).

**Table 5:** Confirmatory factor analysis (CFA) fit indices.

Fit index	Criterion	Measured value	Evaluation conclusion
Chi-square/df (*χ*^2^/*df*)	<3.0 or < 5.0	2.846	Good
RMSEA	<0.08	0.068	Good
CFI	>0.90	0.952	Excellent
TLI	>0.90	0.947	Excellent
SRMR	<0.08	0.041	Excellent

The confirmatory factor analysis results show that the measurement model generally fits well: *χ*^2^/*df* = 2.846, RMSEA = 0.068, CFI = 0.952, TLI = 0.947, SRMR = 0.041, all meeting common judgment standards. This result indicates that the latent variable structure set in this paper can well fit the current sample data, providing a certain support for the subsequent structural relationship tests.

#### 4.3.3 Discriminant validity and variable identification

To test the degree of discrimination among core latent variables, this study judged discriminant validity by comparing Pearson correlation coefficients with the square roots of AVE. According to the criterion proposed by Fornell and Larcker (1981), if the square root of the AVE of each construct is greater than its correlation coefficients with other variables, it indicates that the latent variable has good discriminant validity. Specific results are shown in Table 6.

**Table 6:** Correlation coefficient matrix and discriminant validity test.

Variable	1	2	3
1. Intergenerational mentoring	(**0.787**)		
2. Human capital perception	0.524***	(**0.696**)	
3. Receptivity to change	0.665***	0.578***	(**0.764**)

The bold values in brackets on the diagonal are the square roots of the AVEs for each latent variable; ***p < 0.001.

Table 6 shows that all three core variables are significantly positively correlated (*p* < 0.001). From the perspective of discriminant validity, the square roots of AVEs for each latent variable (0.787, 0.696, and 0.764 respectively) are strictly greater than the maximum correlation values between them and the other variables in the corresponding row/column (0.665). This result conforms to the Fornell-Larcker criterion, indicating that the three core constructs have good discriminant validity in the current sample. This solid measurement foundation provides a strong guarantee for subsequently testing the structural paths of independent variables and mediating variables.

### 4.4 Hypothesis testing: statistical mediation association analysis

#### 4.4.1 Hierarchical regression analysis

To test the statistical mediation association of human capital perception between intergenerational mentoring characteristics and receptivity to change, this study adopted hierarchical multiple regression analysis for preliminary testing. Intergenerational mentoring characteristics are jointly composed of three dimensions: career and skill support, psychosocial support, and role modeling. Receptivity to change acts as the dependent variable, human capital perception as the mediating variable, and gender and tenure are simultaneously included in the model as control variables. Specific analysis results are shown in Table 7.

**Table 7:** Hierarchical regression analysis results for mediation effects.

Variable	Model 1: Change receptivity	Model 2: Human capital perception	Model 3: Change receptivity
Control variables			
Gender	0.007 (0.024)	−0.060 (0.040)	0.023 (0.022)
Tenure	0.005 (0.009)	0.034 (0.016)	−0.004 (0.008)
Independents and mediators			
Intergen. mentoring	0.677*** (0.027)	0.657*** (0.037)	0.507*** (0.029)
Human capital percep.	—	—	0.259*** (0.023)
Model testing			
*R* ^2^	0.443	0.280	0.515
Adjusted *R*^2^	0.441	0.278	0.513
*F*-value	213.910***	104.867***	214.350***

Values in the table are unstandardized regression coefficients B, standard errors (SE) are in parentheses; model calculations include the constant term; ***p < 0.001.

Before formally interpreting regression results, this study conducted multi-collinearity diagnostics on the model. The results showed that the tolerance of each variable ranged from 0.719 to 0.994, and the maximum VIF value was 1.389, all within acceptable ranges, indicating no serious multicollinearity issues exist in the model.

Table 7 shows that after controlling for gender and tenure, intergenerational mentoring characteristics are significantly positively associated with employees’ receptivity to change (Model 1: *B* = 0.677, *β* = 0.665, *p* < 0.001), supporting H1. Model 2 shows that intergenerational mentoring characteristics are significantly positively associated with human capital perception (*B* = 0.657, *β* = 0.527, *p* < 0.001), supporting H2. Model 3 shows that after simultaneously including intergenerational mentoring characteristics and human capital perception, human capital perception is still significantly positively associated with receptivity to change (*B* = 0.259, *β* = 0.318, *p* < 0.001), supporting H3; concurrently, the regression coefficient of intergenerational mentoring characteristics decreases from *B* = 0.677 to *B* = 0.507 (with the standardized coefficient *β* decreasing from 0.665 to 0.498, both *p* < 0.001), and the model’s overall explanatory power (*R*^2^) increases from 0.443 to 0.515. This Δ*R*^2^ of 0.073 yields an incremental effect size of approximately *f*^2^ = 0.150, indicating a moderate and practically meaningful additional explanatory contribution from the mediator. These results imply that human capital perception presents a significant partial statistical mediation association between the two variables, preliminarily supporting H4.

#### 4.4.2 Bootstrap mediation test

To further test the mediating role of human capital perception, this study refers to Hayes (2017) suggestions and uses the Bootstrap method for resampling tests, with 5,000 resamples, and calculates the 95% confidence interval of the indirect effect. Specific results are shown in Table 8.

**Table 8:** Bootstrap mediation effect test results.

Mediation path	Effect size	95% Confidence interval	Effect ratio	Test conclusion
Total effect	0.677	[0.618, 0.736]	100%	Significant
Direct effect	0.507	[0.450, 0.564]	74.8%	Significant
Indirect effect via human capital perception	0.171	[0.135, 0.206]	25.2%	Significant

Effect sizes are unstandardized effects; confidence intervals not containing 0 indicate the effect has reached a statistical significance level.

Bootstrap results show that the indirect effect via human capital perception is significant, with an indirect effect of *B* = 0.171, and a 95% confidence interval of [0.135, 0.206], which does not contain 0. The direct effect is *B* = 0.507, and the 95% confidence interval is [0.450, 0.564], which also does not contain 0. The indirect effect accounts for 25.2% of the total effect. This indicates that contextualized human capital perception explains a meaningful but partial share of the association between intergenerational mentoring and change receptivity, while most of the association remains direct or may operate through other unmeasured mechanisms.

**Figure 3:**
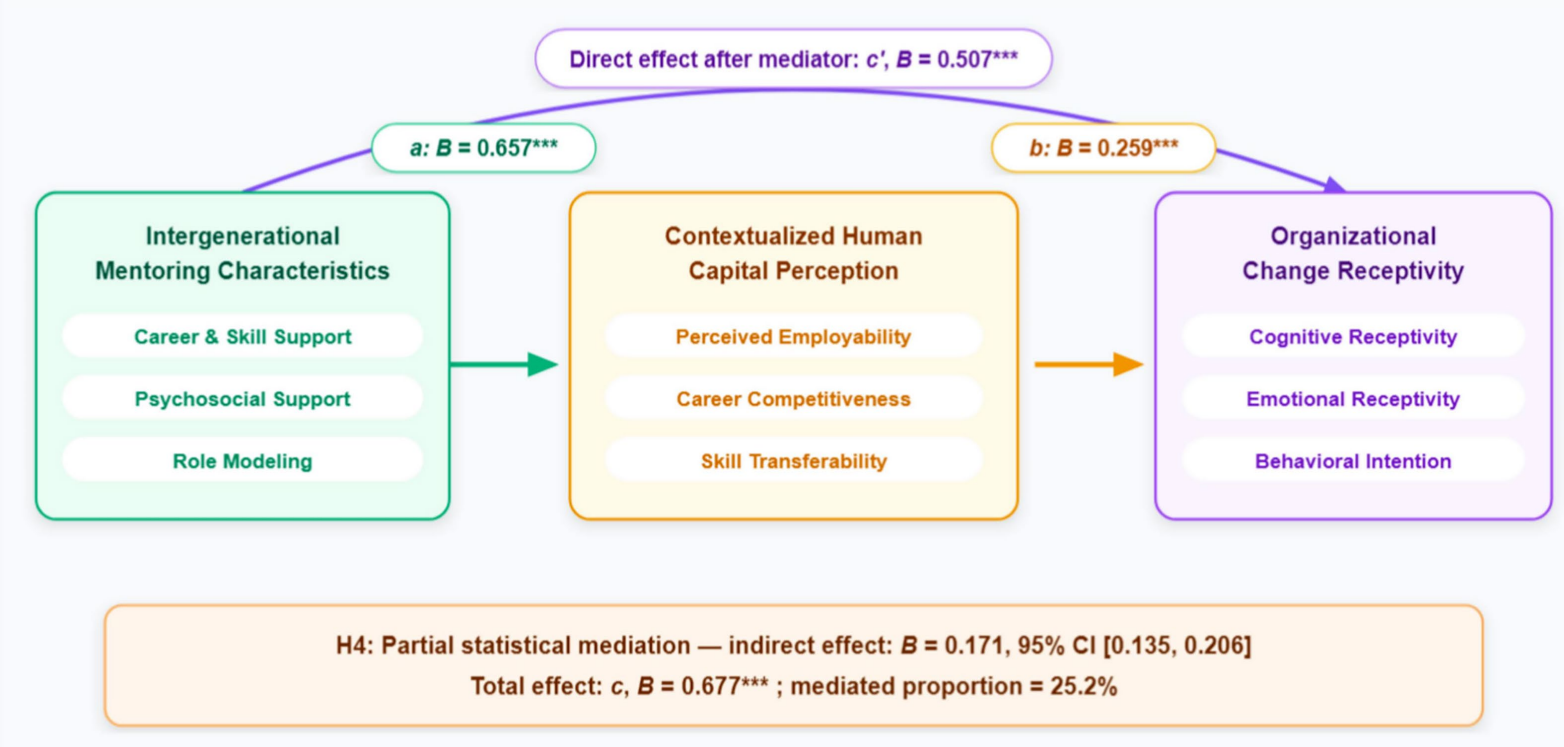
Partial statistical mediation effect path diagram of human capital perception. The figure reports unstandardized regression coefficients *B*; path coefficients *a*, *b*, *c*′ come from hierarchical regression analysis, and the indirect effect and its 95% confidence interval come from 5,000 Bootstrap resampling tests. Gender and tenure are included in the model as control variables but are not shown in the diagram. *** *p* < 0.001.

To more intuitively present the statistical mediation path among intergenerational mentoring characteristics, human capital perception, and organizational change receptivity, this study plotted Figure 3 based on hierarchical regression and Bootstrap test results. In the figure, path *a* represents the positive association between intergenerational mentoring characteristics and human capital perception, path *b* represents the positive association between human capital perception and organizational change receptivity, *c*′ represents the direct effect still existing between intergenerational mentoring characteristics and organizational change receptivity after including the mediating variable, and *c* represents the total effect. Results show that paths *a*, *b*, and *c*′ all reach significance levels, and the confidence interval of the Bootstrap indirect effect does not contain 0, indicating that human capital perception presents a significant partial statistical mediation association between the two.

The above test evidence indicates that human capital perception presents a significant partial statistical mediation association between intergenerational mentoring characteristics and organizational change receptivity, and hypothesis H4 is supported. In practical terms, this means that a good intergenerational interaction network not only co-occurs directly with a more positive attitude towards change but also establishes an indirect statistical association with receptivity to change through the self-career competitiveness advantages perceived by employees. Given the cross-sectional design, this pattern should be interpreted as statistical mediation rather than temporal causality.

### 4.5 Heterogeneity test of demographic characteristics

To answer the exploratory question regarding birth generation differences (RQ1) and to complementarily examine whether other objective demographic characteristics were associated with attitudinal differentiation among employees, this study performed between-group difference tests using birth generation, rank, and industry as grouping variables. Before the analysis, the study recoded rank into two categories: “grassroots employees” and “management” to simplify the category structure and improve between-group comparability. Levene’s test for equality of variances showed that all *p*-values were greater than 0.05, meeting the premise assumptions of parametric tests. Specific analysis results are shown in Table 9.

**Table 9:** Analysis of differences in receptivity to change across demographic characteristics (*N* = 812).

Classification	Groupings	*N*	*M*	*SD*	Test statistic	*p*-value
Birth generation	1. Senior generation (≤1979)	36	3.644	0.448	*F*(3,808) = 0.301	0.825
	2. Post-80s (1980–1989)	271	3.624	0.428		
	3. Post-90s (1990–1999)	379	3.665	0.435		
	4. Post-00s New Gen (≥2000)	126	3.641	0.404		
Current rank	Grassroots employees	446	3.638	0.413	*t*(810) = –0.520	0.603
	Management	366	3.658	0.446		
Industry	1. Internet and communication tech	209	3.641	0.438	*F*(5,806) = 0.338	0.890
	2. Finance and investment capital	116	3.670	0.416		
	3. Manufacturing and engineering	247	3.644	0.415		
	4. Medical and pharmaceutical health	60	3.623	0.422		
	5. Retail services and consumer goods	127	3.634	0.463		
	6. Other industries	53	3.679	0.416		

Test statistics correspond to the ANOVA *F*-value and independent samples *t*-test *t*-value, respectively. The rank variable was recoded into two categories: grassroots employees and management. First-line, middle, and top management positions were merged into the management category.

**Figure 4:**
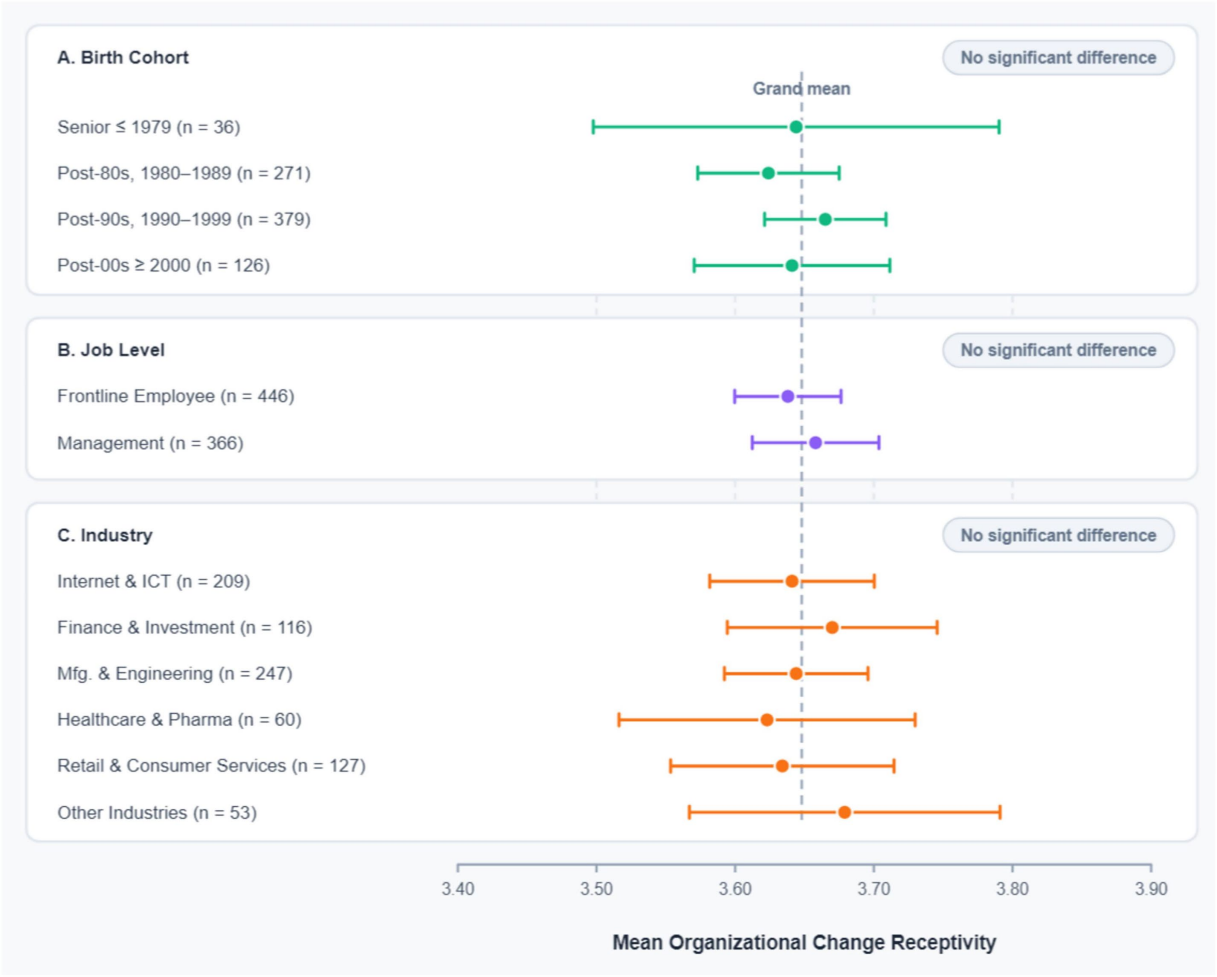
Means and 95% confidence intervals of organizational change receptivity for different demographic groups. The dots in the figure represent the mean score of organizational change receptivity for each group, and the horizontal error bars represent the 95% confidence intervals; the dashed line represents the overall sample mean. The figure is for visual display only; specific means, standard deviations, and significance test results are shown in Table 9.

To more intuitively visualize the distributional differences in organizational change receptivity among different demographic groups, this study further plotted Figure 4. The dashed line in the figure represents the overall sample mean of change receptivity. The distribution of mean points and confidence intervals across groups is quite close, which is consistent with the significance test results in Table 9, indicating that no significant between-group differences were found for birth generation, rank, and industry groupings.

Combining the significance tests in Table 9 and the visualization results in Figure 4, the exploratory test proposed for RQ1 and the supplementary heterogeneity analysis results reveal no significant group differences across three core attributes:

At the birth generation level, the ANOVA test results (*F* = 0.301, *p* = 0.825) show that the receptivity to change among employees from different generations is at a moderate-to-high level (means ranging from 3.62 to 3.67), finding no significant between-group differences for the exploratory test of RQ1. The extremely weak effect size (*η*^2^ ≈ 0.001) further suggests that generational labels explained almost zero variance in employees’ receptivity to organizational change. However, this non-significant result should not be interpreted as definitive evidence that generational differences never exist. The senior-generation group contained only 36 participants, and the group sizes were unequal, which may reduce statistical power for detecting small differences. Therefore, the finding is better understood as showing that no meaningful generational difference was detected in the present sample, rather than as proving complete equivalence across generations.

At the supplementary level of rank and industry, the independent samples *t*-test indicated no significant attitudinal deviation between grassroots employees and management (*t* = −0.520*, p* = 0.603); the ANOVA also showed that the difference in receptivity among employees from different industrial backgrounds was statistically insignificant (*F* = 0.338*, p* = 0.890), with an effect size of approximately *η*^2^ ≈ 0.002. This suggests that even though management usually holds more change-related information, or different industries face different digital pressures, these macro-identity labels showed limited explanatory power for employees’ mentality toward embracing change.

Overall, objective demographic indicators such as birth generation, rank, and industry background showed weak explanatory efficacy for change receptivity in this sample. Rather, the results are consistent with the argument that employees’ receptivity to change may be more closely related to the support resources obtained within their micro-workplace, such as intergenerational mentoring characteristics, and their perceived career-resource security, namely contextualized human capital perception. Nevertheless, because subgroup sizes were unequal and the demographic analyses were exploratory, these findings should be interpreted cautiously and verified in future studies using more balanced cohort samples.

## 5 Conclusion and discussion

### 5.1 Research conclusions

Based on the realistic background of normalized organizational change and the coexistence of multi-generational employees, this study took 812 practitioners as a sample to examine the association between intergenerational mentoring characteristics, contextualized human capital perception, and organizational change receptivity. The study primarily draws the following three conclusions:

First, intergenerational mentoring characteristics are significantly and positively correlated with employees’ organizational change receptivity. The more cross-generational career skill support, psychosocial support, and role modeling influence employees perceive in the workplace, the more positive their attitude of acceptance and cooperation in coping with change tends to be. This finding is consistent with the view that cross-generational interaction networks constitute important relational support resources in organizational change contexts (Kordova et al., 2022; Tang and Martins, 2021).

Second, contextualized human capital perception showed a significant partial statistical mediation association between intergenerational mentoring characteristics and organizational change receptivity. This indicates that intergenerational mentoring networks are not only directly related to receptivity to change, but also establish an indirect statistical association by positively shaping employees’ subjective assessments of their own career competitiveness, job adaptability, and external market value. However, this conclusion should be understood as an associational pattern based on cross-sectional data rather than as evidence of a confirmed causal sequence (Ho et al., 2023; Malik and Nawaz, 2022).

Third, in this sample, employees from different birth generations, positional ranks, or industries showed no significant between-group differences in their receptivity to change. This finding suggests that when facing digitalization and process restructuring, differences in employees’ change attitudes might not be primarily determined by macro demographic or industry labels. Nevertheless, this result should be interpreted as sample-specific, especially given the relatively small senior-generation subgroup and unequal group sizes, rather than as definitive evidence that generational differences never exist (Alaql et al., 2023; Kim and Jung, 2022).

### 5.2 Theoretical contributions

This study makes theoretical expansions to the research on organizational behavior and multi-generational employee management in the following three aspects: First, this paper introduces intergenerational mentoring interactions into the relevant influencing factors of organizational change receptivity, enriching the research on the micro-mechanisms of organizational change. Differentiating from existing literature highly focused on top-down institutional factors like transformational leadership and formal communication, this paper suggests that localized and reverse support (skills, psychology, and role models) among cross-generational colleagues is equally a key micro-resource for building employees’ positive attitudes towards change (Browne, 2021; Khaw et al., 2023). Second, this paper introduces the contextualized human capital perception perspective, bridging the psychological explanatory pathway of “cross-generational interaction — individual resource perception — change attitude.” Rather than reifying human capital as only an objective stock of education, certificates, or tenure, this study focuses on employees’ subjective appraisal of whether their career-related resources remain useful, transferable, and valuable under organizational change. This perspective helps explain why intergenerational mentoring may be associated with more positive change attitudes: cross-generational support provides relational resources, while employees’ perceived career-resource sufficiency reduces the sense of resource threat. In this sense, human capital theory defines the focal resource, social exchange theory explains the relational source of that resource, and conservation of resources theory explains why perceived resource sufficiency is associated with receptivity to change (Blau, 1964; Cropanzano and Mitchell, 2005; Hobfoll, 1989).

Finally, the heterogeneity test results provide an empirical reflection on workplace generational stereotypes. The study found no significant differences in change receptivity across birth cohorts in the present sample, suggesting that resistance to change should not be mechanically attributed to age labels. This interpretation is consistent with recent organizational psychology research cautioning against treating generational categories as fixed and deterministic predictors of workplace attitudes and behaviors (Rudolph et al., 2021). This finding supports a more cautious and resource-based view of multi-generational workplaces: employees at different life stages may share similar concerns about career security when facing turbulent environments. However, because subgroup sizes were unequal, this conclusion should be treated as sample-specific rather than universal (Wang and Duan, 2025).

### 5.3 Management practical implications

At the practical level, this study provides actionable references for enterprises to manage organizational change more flexibly and smoothly:

First, during the change warm-up and implementation stages, enterprises should consciously construct a “cross-generational cross-mentoring” network. Besides relying on administrative orders, HR departments can use informal mechanisms like project-based systems to activate the two-way flow of intergenerational knowledge—both leveraging the stabilizing experience of senior employees and encouraging younger employees to provide digital reverse mentoring. Such mutual assistance may help lower operational obstacles and interpersonal friction in the early stages of change (Li et al., 2024; Venugopal et al., 2025).

Second, the core of change communication is not just about painting an “organizational vision,” but also about implementing “individual empowerment.” Since contextualized human capital perception appears to be a key statistical mediating bridge, managers are advised to help employees genuinely perceive, through certification training, mentoring, and career planning, that organizational change may also provide opportunities to expand cognitive boundaries and accumulate external market competitiveness (Jackson et al., 2024; Lo Presti et al., 2022).

Third, abandon empirical intergenerational management stereotypes. Since older employees are not necessarily naturally resistant, and the new generation may not completely embrace it, enterprises should avoid adopting simplified or marginalizing treatments targeted at specific senior groups when formulating change appeasement strategies. Instead, a comprehensive resource support system covering all staff should be established to jointly mitigate the capability anxieties under changing circumstances (Kim and Jung, 2022; Walton, 2024).

## 6 Research limitations and future prospects

Although theoretical and empirical analyses support the proposed statistical associations, this study still has several limitations that should be addressed in future research:

First, the cross-sectional design limits causal inference. The core data of this study was entirely measured at a single time point. Even though the results align with the theoretically presupposed logical associations, they cannot provide strong temporal causal proof. It is also theoretically possible that employees who are naturally more receptive to change are more likely to proactively seek and engage in intergenerational mentoring. Future research is urged to adopt a longitudinal tracking design, collecting data in stages of organizational change to dynamically examine how cross-generational interactions and employees’ change attitudes co-evolve over time.

Second, the risk of common method bias from single-source self-reported data cannot be completely eliminated. Constrained by practical operations, this paper mainly relied on employees’ self-reports. Although this study employed procedural remedies, Harman’s single-factor test, and full collinearity VIF diagnostics to rule out severe common method bias, residual common method variance cannot be fully excluded because no theoretically unrelated marker variable was included in the questionnaire. Future empirical designs could introduce multi-source matching mechanisms, such as supervisor-rated change cooperation behaviors or peer-rated intergenerational support quality, to enhance measurement objectivity.

Third, the operationalization of contextualized human capital perception requires further refinement. The scale used in this paper was adapted to the organizational change context, and some items may have semantic proximity to either intergenerational communication or change-related skill development. Furthermore, because this study used self-perceived employability indicators to capture employees’ subjective resource appraisals, the scale may reflect perceived employability more directly than objective human capital itself. Future research could conduct strict measurement equivalence tests, use bi-factor models, or develop more refined scales to optimize construct distinctiveness.

Fourth, limits related to item parceling and sampling strategy should be acknowledged. Although item-level measurement structures were verified before dimension reduction, parceling may still conceal item-level misspecification or cross-loadings and artificially inflate model fit indices. Additionally, because the study relied on non-probability convenience sampling and online snowball recruitment, potential self-selection bias cannot be fully ruled out. Unequal subgroup sizes, especially in demographic comparison analyses, also limit the generalizability of non-significant group-difference findings.

Finally, there remains insufficient control over sample-context heterogeneity. In measuring the dependent variable, some respondents based their answers on their “most memorable” past change memories, which might mix in systematic noise regarding event intensity and recall timeliness. Subsequent research could try tracking specific groups currently undergoing the exact same specific change event (e.g., uniform digital cloud migration of a large enterprise), and further incorporate trait variables such as individual psychological resilience into boundary condition considerations (Oreg, 2006), thereby establishing a fuller and more rigorous theoretical explanatory framework.

## Data Availability

The original contributions presented in the study are included in the article/Supplementary material, further inquiries can be directed to the corresponding author/s.
