# Supplementary material for: An analysis of the association between workplace intergenerational mentoring characteristics and employees’ receptivity to change: the mediating role of contextualized human capital perception
Source: Front Psychol. 2026 Jul 27;17:1899021. doi: 10.3389/fpsyg.2026.1899021 (PMC13453695; doi:10.3389/fpsyg.2026.1899021)
Supplement: Supplementary file 1 [file Data_Sheet_1.ZIP › Supplementary Material 2 Data Codebook.docx]

### 1. 结构与变量命名规则 (Structure and Naming Convention)

**IV (Independent Variable自变量):** Mentor (Intergenerational Mentoring Characteristics / 代际导师特征)

**DV (Dependent Variable因变量):** Change (Attitude Toward Change / 变革态度)

**Med (Mediator 中介变量): HC (Contextualized Human Capital Perception / 情境化人力资本感知)**

**Reverse-scored items (反向计分题):** Marked with an asterisk (*). 需要执行重新编码 (1=5, 2=4, 3=3, 4=2, 5=1).

### 2. 人口统计学与控制变量 (Demographics & Control Variables)

| Variable Name (变量名) | Question (对应题目) | Variable Label (变量标签) | Coding Scheme & Value Labels (编码规则与标签) | Data Measure (测量尺度) |
| --- | --- | --- | --- | --- |
| Screen18 | Q1 | ≥18 years old & Employed (年满18且在职甄别) | 1 = Yes (是); 2 = No (否) *[Data omitted / 剔除样本]* | Nominal (定类) |
| Gender | Q2 | Gender (性别) | 1 = Male (男); 2 = Female (女) | Nominal (定类) |
| GenCohort | Q3 | Generational Cohort (代际分组) | 1 = Pre-1979 (X世代及以前); 2 = 1980-1989 (80后); 3 = 1990-1999 (90后); 4 = 2000-2006 (00后) | Ordinal (定序) / Nominal |
| Industry | Q4 | Industry Sector (所属行业) | 1 = IT/Telecom (互联网/通信); 2 = Finance (金融); 3 = Manufacturing (制造); 4 = Healthcare (医疗); 5 = Retail/Service (零售/服务); 6 = Others (其他) | Nominal (定类) |
| Tenure | Q5 | Tenure in Organization (本企业任职年限) | 1 = <1 year (1年内); 2 = 1-3 years (1-3年); 3 = 3-5 years (3-5年); 4 = 5-10 years (5-10年); 5 = >10 years (10年以上) | Ordinal (定序) |
| Position | Q6 | Job Level (职级状态) | 1 = Junior Staff (基层员工); 2 = First-line Mgr (基层管理); 3 = Middle Mgr (中层管理); 4 = Senior Exec (高层管理) | Ordinal (定序) |
| ChangeExp | Q7 | Recent Change Experience (近期变革经历) | 1 = Yes (是); 2 = No (否) | Nominal (定类) |

### 3. 李克特量表核心变量 (Likert-Scale Core Variables)

**Coding Scheme (全局编码规则):**
1 = Strongly Disagree (完全不同意); 2 = Disagree (比较不同意); 3 = Neutral (不确定/一般); 4 = Agree (比较同意); 5 = Strongly Agree (完全同意)

#### 自变量：代际导师特征 (IV: Intergenerational Mentoring Characteristics)

| Variable Name (变量名) | Question (题目) | Sub-dimension (所属子维度) | Variable Label (变量具体内容标签) |
| --- | --- | --- | --- |
| Mentor_CS1 | 8-1 | Career/Skill Support | Shares skills/new systems (分享技能/新系统) |
| Mentor_CS2 | 8-2 | (职业与技能支持) | Provides coaching on tasks (提供任务辅导) |
| Mentor_CS3 | 8-3 |  | Expands knowledge base (扩充知识储备) |
| Mentor_CS4 | 8-4 |  | Explains mechanisms/resources (解释机制/隐性资源) |
| Mentor_CS5 | 8-5 |  | Sponsors/Recommends for projects (赞助/引荐项目) |
| Mentor_PS1 | 8-6 | Psychosocial Support | Shares anxiety/setbacks (分享焦虑/挫折) |
| Mentor_PS2 | 8-7 | (心理与社会支持) | Conveys trust/encouragement (传递信任/鼓励) |
| Mentor_PS3 | 8-8 |  | Understands emotions/stress (理解情绪/压力) |
| Mentor_PS4 | 8-9 |  | Trustworthy listener (值得信赖的倾听者) |
| Mentor_PS5 | 8-10 |  | Feels respected/accepted (感到受尊重与接纳) |
| Mentor_RM1 | 8-11 | Role Modeling | Appreciates work attitude (欣赏工作态度) |
| Mentor_RM2 | 8-12 | (角色榜样与角色认同) | Reference for behavior (行为参考点) |
| Mentor_RM3 | 8-13 |  | Learns from generational traits (学习时代特征沉淀) |
| Mentor_RM4 | 8-14 |  | Identifies with values/ethics (认同价值观/道德) |
| Mentor_RM5 | 8-15 |  | Emulates complex skills (模仿复杂沟通技巧) |

#### 因变量：组织变革态度评价 (DV: Attitude Toward Organizational Change)

| Variable Name (变量名) | Question (题目) | Sub-dimension (所属子维度) | Variable Label (变量具体内容标签) |
| --- | --- | --- | --- |
| Change_CR1 | 9-1 | Cognitive Receptivity | Necessary and beneficial (必要且有益的) |
| Change_CR2 | 9-2 | (变革认知度) | Understands business logic (理解商业逻辑) |
| Change_CR3 | 9-3 |  | Risks of not changing (不改变的风险) |
| Change_CR4 | 9-4 |  | Improves ultimate performance (最终提升效能) |
| Change_CR5_R* | 9-5 |  | [Reverse] Completely unnecessary (完全多此一举) |
| Change_ER1 | 9-6 | Emotional Receptivity | Optimistic and hopeful (乐观与充满希望) |
| Change_ER2 | 9-7 | (变革情感状态) | Exciting learning process (令人兴奋的学习过程) |
| Change_ER3 | 9-8 |  | Maintains inner peace (内心保持平静) |
| Change_ER4_R* | 9-9 |  | [Reverse] Highly anxious (感到非常焦虑) |
| Change_ER5_R* | 9-10 |  | [Reverse] Threats to job security (感受到利益受威胁) |
| Change_BI1 | 9-11 | Behavioral Intentions | Follows new processes (主动遵循新流程) |
| Change_BI2 | 9-12 | (变革行为意向) | Explains benefits to others (向抱怨者解释好处/安抚) |
| Change_BI3 | 9-13 |  | Exerts extra effort/time (付出额外精力/时间帮助团队) |
| Change_BI4 | 9-14 |  | Provides constructive feedback (积极提供改进建议) |
| Change_BI5 | 9-15 |  | Invests personal time to learn (投入私人时间学习新技能) |

#### 中介/效标变量：个人发展感知 (Med/Consequence: Human Capital Perception)

| Variable Name (变量名) | Question (题目) | Sub-dimension (单维度) | Variable Label (变量具体内容标签) |
| --- | --- | --- | --- |
| HC_1 | 10-1 | Human Capital | Improved core competitiveness (提升核心竞争力) |
| HC_2 | 10-2 | (人力资本感知) | Adaptability as invisible asset (适应力是隐形资产) |
| HC_3 | 10-3 |  | Increased external market value (外部市场价值增加) |
| HC_4 | 10-4 |  | Broadened cognitive boundaries (认知边界拓宽与敏捷) |
| HC_5 | 10-5 |  | Valuable career investment (长期职业生涯的投资) |
