# Supplementary material for: An analysis of the association between workplace intergenerational mentoring characteristics and employees’ receptivity to change: the mediating role of contextualized human capital perception
Source: Front Psychol. 2026 Jul 27;17:1899021. doi: 10.3389/fpsyg.2026.1899021 (PMC13453695; doi:10.3389/fpsyg.2026.1899021)
Supplement: Supplementary file 1 [file Data_Sheet_1.ZIP › Supplementary Material 1 Survey Questionnaire.docx]

### Informed Consent & Ethical Statement (知情同意与伦理声明)

**Dear participant (尊敬的受访者):**
We are an academic research team focusing on management and organizational behavior. Currently, various industries are facing different degrees of business or management transformations. We aim to understand the authentic experiences of employees from different generations regarding workplace interactions and their receptivity to organizational change.
我们是进行管理学与组织行为学研究的学术团队。当前，各行各业面临管理转型，我们希望了解不同年龄段员工在职场互动与应对变革时的真实感受。

**Ethical Declaration:** Participation requires you to be at least 18 years old and currently employed. Responses are strictly anonymous and will only be used for academic statistical analyses.
根据学术伦理规范，参与者须年满18周岁且当前在职。本问卷实行绝对匿名，数据仅用于学术统计分析。

**Concept Definition:** The term “Mentor/Colleague” in this survey refers to a coworker from a different generation who has guided you, including senior employees guiding junior ones, or young employees guiding senior ones (reverse mentoring).
概念提示：“职场导师/同事”既包括指导您的资深老员工，也包括在技术上指导您的年轻同事（逆向导师）。

### **Part I: Demographic & Screening Information (第一部分：基本信息与甄别)**

**1. Are you at least 18 years old? (您是否已满18周岁？)**

Yes, I am 18 or older and currently employed. (是，我已满18周岁并在职)

No, I am under 18 years old. *(Survey terminates)* (否，我未满18周岁)[终止作答]

**2. Gender (性别):**

Male (男) / [ ] Female (女)

**3. Generational Cohort / Birth Year (您的出生年代/代际):**

1979 or earlier / Baby Boomers & Gen X (1979年及以前)

1980 - 1989 / Gen Y - Early Millennials (80后一代)

1990 - 1999 / Gen Y - Late Millennials (90后一代)

2000 - 2006 / Gen Z (00后一代)

**4. Industry of your current organization (您所在企业所属的行业):**

IT/Telecommunication (互联网/信息技术/通信)

Finance/Insurance/Investment (金融/保险/投资)

Manufacturing/Engineering (制造业/实业/工程)

Healthcare/Pharmaceuticals (医疗/生物/制药)

Retail/Wholesale/Services (批发/零售/服务业)

Others (其他)

**5. Tenure in the current organization (在目前企业的任职年限):**

< 1 year (1年以内) / [ ] 1-3 years (1-3年) / [ ] 3-5 years (3-5年) / [ ] 5-10 years (5-10年) / [ ] > 10 years (10年以上)

**6. Current Position (您的职级状态):**

Junior Staff (基层员工，无下属)

First-line Manager (基层管理，如主管/组长)

Middle Manager (中层管理，如部门经理)

Senior Executive (高层管理)

**7. Has your organization experienced or is currently undergoing significant management/system changes in the past year? (近一年内，公司是否在经历显著的管理或制度变革？)**

Yes (是，正在经历或刚经历)

No *(Please answer subsequent questions based on the most profound organizational change in your past career)* (否，请基于过往最深刻的一次变革经历作答)

### **Part II: Intergenerational Mentoring Characteristics (第二部分：跨代际互动与指导特征)**

**Instruction:** Please recall a colleague from a different generation who has guided/mentored you. Evaluate the following statements. (请回想给您提供过指导的跨代际同事，根据交流感受评分。)
*(1 = Strongly Disagree 完全不同意; 3 = Neutral 不确定/一般; 5 = Strongly Agree 完全同意)*

| No. | Statement (评估陈述) | 1 | 2 | 3 | 4 | 5 |
| --- | --- | --- | --- | --- | --- | --- |
| 8-1 | This colleague frequently shares professional skills or knowledge regarding new systems with me. (他/她经常向我分享完成某项具体工作或使用新系统的专业技能。) | ○ | ○ | ○ | ○ | ○ |
| 8-2 | They provide coaching when I take on challenging tasks or adapt to new business operations. (他/她会在我负责更具挑战性的任务时提供业务辅导。) | ○ | ○ | ○ | ○ | ○ |
| 8-3 | This intergenerational interaction has significantly expanded my professional knowledge base. (这种跨代际的交流，显著扩充了我的专业知识储备。) | ○ | ○ | ○ | ○ | ○ |
| 8-4 | Their guidance helped me better understand the internal mechanisms and hidden resources of the company. (他/她的指导帮助我更好地了解了公司内部的运作机制与隐性资源。) | ○ | ○ | ○ | ○ | ○ |
| 8-5 | They have helped me gain opportunities to showcase my abilities or recommended me for important projects. (他/她曾帮我争取过展示能力的机会，或引荐我参与重要项目。) | ○ | ○ | ○ | ○ | ○ |
| 8-6 | I am willing to share with them when I feel anxious or face interpersonal setbacks at work. (当我在工作中感到焦虑或遇到人际挫折时，我愿意与他/她分享。) | ○ | ○ | ○ | ○ | ○ |
| 8-7 | They convey trust and encouragement regarding my personal abilities during our communications. (他/她会在交流中传递出对我个人能力的信任和鼓励。) | ○ | ○ | ○ | ○ | ○ |
| 8-8 | Despite the age difference, they understand my emotions and stress in the workplace. (尽管年龄/代际不同，他/她依然能够理解我在职场中的情绪与压力。) | ○ | ○ | ○ | ○ | ○ |
| 8-9 | To me, they are not just coworkers but trustworthy listeners in the workplace. (他/她对我而言不仅是同事，更是值得信赖的职场倾听者。) | ○ | ○ | ○ | ○ | ○ |
| 8-10 | I often feel respected and accepted in my discussions with them. (在和他/她的探讨中，我常常感到自己是被尊重和接纳的。) | ○ | ○ | ○ | ○ | ○ |
| 8-11 | I deeply appreciate their attitude toward work and their approach to problem-solving. (我很欣赏他/她对待工作的态度和解决问题的方式。) | ○ | ○ | ○ | ○ | ○ |
| 8-12 | Their behavior serves as an excellent reference point for my future workplace performance. (他/她的行为方式为我今后的职场表现提供了很好的参考点。) | ○ | ○ | ○ | ○ | ○ |
| 8-13 | They possess generational traits or accumulated experience that I lack and are worth learning from. (他/她身上具备某些我缺乏的时代特征或经验沉淀，值得我学习。) | ○ | ○ | ○ | ○ | ○ |
| 8-14 | I strongly identify with the profound work ethic and values they demonstrate. (他/她展现出的职业道德和价值观，令我深感认同。) | ○ | ○ | ○ | ○ | ○ |
| 8-15 | I consciously emulate their skills in cross-departmental communication or managing complex issues. (我会有意识地模仿他/她在跨部门沟通等方面的技巧。) | ○ | ○ | ○ | ○ | ○ |

### Part III: Attitude Toward Organizational Change (第三部分：组织变革态度评价)

Instruction: Please evaluate your true attitude regarding the recent organizational change mentioned in Question 7. (请结合上文提及的公司近期变革，评价您的真实态度。)
*(1 = Strongly Disagree 完全不同意; 5 = Strongly Agree 完全同意;* ***[R] indicates Reverse-scored item [R]代表反向计分题****)*

| No. | Statement (评估陈述) | 1 | 2 | 3 | 4 | 5 |
| --- | --- | --- | --- | --- | --- | --- |
| 9-1 | I believe implementing this change is necessary and beneficial for the company’s future. (我认为开展这项变革对企业未来的发展是必要且有益的。) | ○ | ○ | ○ | ○ | ○ |
| 9-2 | I clearly understand the business logic behind the management’s push for this change. (我能够清晰地理解管理层推行这项变革的商业逻辑。) | ○ | ○ | ○ | ○ | ○ |
| 9-3 | In the long run, the company will face greater risks if it does not change. (从长远来看，不改变的话公司将面临更大的风险。) | ○ | ○ | ○ | ○ | ○ |
| 9-4 | I believe this change will ultimately improve team or individual performance. (我相信这项变革最终能提升团队或个人的工作效能。) | ○ | ○ | ○ | ○ | ○ |
| 9-5 | [R] I think the company’s implementation of this change is completely unnecessary. (我觉得公司推行这项变革完全是多此一举。) | ○ | ○ | ○ | ○ | ○ |
| 9-6 | Overall, I feel optimistic and hopeful about this organizational change. (面对这项组织变革，我总体上感到乐观和充满希望。) | ○ | ○ | ○ | ○ | ○ |
| 9-7 | Adapting to these changes is an exciting learning process rather than a burden to me. (对我而言，适应新变化是令人兴奋的学习过程，而非负担。) | ○ | ○ | ○ | ○ | ○ |
| 9-8 | Even amidst early confusion from the change, I maintain relative inner peace. (即使变革初期出现混乱，我内心也能保持相对的平静。) | ○ | ○ | ○ | ○ | ○ |
| 9-9 | [R] I feel highly anxious when thinking about recent changes in company systems. (想到近期发生的这些变化，我就感到非常焦虑。) | ○ | ○ | ○ | ○ | ○ |
| 9-10 | [R] This change makes me feel that my job security or vested interests are threatened. (这次变革让我感到岗位安全或固有利益受威胁。) | ○ | ○ | ○ | ○ | ○ |
| 9-11 | I am willing to actively try and follow the new processes/systems in daily work. (日常工作中，我愿意主动尝试并遵循变革后的新流程。) | ○ | ○ | ○ | ○ | ○ |
| 9-12 | When colleagues complain about the change, I tend to explain its benefits or calm them down. (当同事抱怨时，我倾向于解释好处或安抚他们。) | ○ | ○ | ○ | ○ | ○ |
| 9-13 | I am willing to exert extra effort and time to help the team transition smoothly. (我愿意付出额外精力，帮助团队更平稳地度过转型期。) | ○ | ○ | ○ | ○ | ○ |
| 9-14 | I am willing to provide constructive feedback rather than passively resisting when flaws arise. (出现漏洞时，我愿意积极提出改进意见，而非消极抵抗。) | ○ | ○ | ○ | ○ | ○ |
| 9-15 | I am willing to invest personal time to learn the new skills required for this change. (我愿意投入私人时间来学习这次变革所需的新技能。) | ○ | ○ | ○ | ○ | ○ |

### Part IV: Human Capital / Competitiveness Perception (第四部分：人力资本/个人发展感知)

**Instruction:** Reflecting on the mentoring you received and the organization’s changes, how do you perceive the changes in your personal competitiveness? (结合指导与变革，您如何看待自身竞争力的变化？)

| No. | Statement (评估陈述) | 1 | 2 | 3 | 4 | 5 |
| --- | --- | --- | --- | --- | --- | --- |
| 10-1 | I feel my core workplace competitiveness has improved after this experience. (经历变革与情境指导后，我感觉核心竞争力提升了。) | ○ | ○ | ○ | ○ | ○ |
| 10-2 | My adaptability to new systems/environments constitutes an invisible career asset. (我对新环境的适应能力构成了隐形的职场资产。) | ○ | ○ | ○ | ○ | ○ |
| 10-3 | The new skills I acquired during this change have increased my value in the external job market. (掌握的新技能增加了我在外部人才市场的价值。) | ○ | ○ | ○ | ○ | ○ |
| 10-4 | Intergenerational communication broadened my cognitive boundaries and made my thinking more agile. (跨代际借交流拓宽了认知边界，使思维更敏捷。) | ○ | ○ | ○ | ○ | ○ |
| 10-5 | I believe the current adaptation pains are a valuable long-term investment in my career. (我相信目前的适应阵痛是对长期职业生涯的宝贵投资。) | ○ | ○ | ○ | ○ | ○ |
